# Performance Evaluation of BD FACSPresto^TM^ Near-Patient CD4 Counter for Monitoring Antiretroviral Therapy in HIV-Infected Individuals in Primary Healthcare Clinics in Thailand

**DOI:** 10.3390/diagnostics12020382

**Published:** 2022-02-02

**Authors:** Kasama Sukapirom, Somrat Matchua, Charin Thepthai, Narinee Srimark, Ladawan Khowawisetsut, Kovit Pattanapanyasat

**Affiliations:** 1 Siriraj Center of Research Excellence in Microparticle and Exosome in Diseases, Research Department, Faculty of Medicine Siriraj Hospital, Mahidol University, Bangkok 10700, Thailand; kasama.suk@mahidol.ac.th (K.S.); narinee.sri@mahidol.ac.th (N.S.); 2 Center of Excellence for Flow Cytometry, Department of Research and Development, Faculty of Medicine Siriraj Hospital, Mahidol University, Bangkok 10700, Thailand; 3 Department of Medical Technology, Chonburi Hospital, Chonburi 20000, Thailand; somrat.p1032@gmail.com; 4 Department of Immunology, Faculty of Medicine Siriraj Hospital, Mahidol University, Bangkok 10700, Thailand; charin.the@mahidol.ac.th; 5 Department of Parasitology, Faculty of Medicine Siriraj Hospital, Mahidol University, Bangkok 10700, Thailand

**Keywords:** AIDS, CD4, FACSPresto, flow cytometry, HIV, point-of-care testing

## Abstract

HIV viral load is more reliable tool for monitoring treatment throughout the course of HIV/AIDS, but the test may be expensive in resource-limited settings. Therefore, enumeration of CD4 T-lymphocyte count remains important in these settings. This study evaluated the performance of BDFACSPresto, a near-patient CD4 counter planned to be used in primary healthcare clinics in Thailand. Results of percent, absolute CD4 count and hemoglobin (Hb) on the FACSPresto were compared with the TriTEST/TruCOUNT/BDFACSCalibur method and a Sysmex hematology analyzer. Phase I of the study was performed in an ISO15189 laboratory. Both percentage and absolute values showed Passing–Bablok slopes within 0.98–1.06 and 0.97–1.13, mean Bland–Altman biases of +1.2% and +20.5 cells/µL, respectively. In phase II, venous and some capillary blood samples were analyzed in four primary healthcare clinics. The results showed good correlation between capillary and venous blood. For venous blood samples, regression lines showed slopes of 1.01–1.05 and 1.01–1.07 for all percentage and absolute values. The overall mean biases were +0.9% and +17.0 cells/µL. For Hb, Passing–Bablok regression result gave slope within 1.01–1.07 and mean bias of −0.06 g/dL. Thus, CD4 enumeration in blood by the FACSPresto is reliable and can be performed to an identical standard at primary healthcare clinics.

## 1. Introduction

Infection by human immunodeficiency virus (HIV) and eventual progression to death of patients in the terminal stage of acquired immune deficiency syndrome (AIDS) remains one of the leading causes of worldwide morbidity and mortality [1,2]. The availability of relatively highly active antiretroviral therapy (ART) has significantly reduced illness and death from HIV infection [3]. However, the treatment regimen is expensive and the cumulative costs of treatment are growing as life expectancy increases. Since the announcement of the ambitious 95-95-95 strategy by the Joint United Nations Program on HIV/AIDS (UNAIDS) in 2014 targeting to end the AIDS epidemic by 2030. The aim was to diagnose 95% of all people living with HIV (PLHIV), provide ART for 95% of those diagnosed and achieve viral suppression for 95% of those who have been treated by 2030 [4]. In Thailand, ART is becoming more accessible to PLHIV, estimates of the PLHIV being treated have been progressively scaled up since the inception of the ART program in 2002, with 80% (lower-upper estimate 70–91%) of a total of 470,000 PLHIV benefitting from this program as of the end of 2017 [5].

Current recommendation tests in developed countries for initiation and monitoring of ART in PLHIV are based on immunological assessment by determining the HIV viral load or enumeration of CD4+ T-lymphocyte count in peripheral blood [6,7]. Although plasma viral load is considered to be more informative for monitoring treatment efficacy and early therapeutic failure throughout the course of HIV/AIDS [8,9], it is expensive and/or not available in resource-constrained countries. CD4 T-lymphocyte count therefore remains the best monitor of a patient’s immune and clinical status, including the risk of opportunistic infections, and informs therapeutic-making for patients with advanced HIV infection [9,10]. Although the conventional flow cytometer remains the accepted standard because of its accuracy, precision, and reproducibility [11,12,13], it is expensive and technically demanding, making it not readily available in most resource-limited settings. An ideal CD4 testing system for rural PLHIV to have convenient access would be a system that is simple to use, cheap, robust, and independent of laboratory infrastructure. Above all, it should reliably provide values for the CD4 T-lymphocyte counts with short turnaround times. To meet these requirements, including its delivery at point-of-care (POC), the BDB FACSPresto™ Near-Patient CD4 counter (Becton Dickinson Biosciences (BDB), San Jose, CA, USA) was developed and commercially launched in 2014 [14]. It is designed to be used in standard voluntary counseling and testing centers normally operated by non-medical personnel. Several studies showed accurate, reliable, precise CD4+ T-lymphocyte and hemoglobin (Hb) results of this new POC system when compared to the reference method irrespective of venous or capillary blood sampling [15,16,17,18,19,20,21]. Nevertheless, most of these studies, reported with varying approaches, predicate platform and outcome, and were performed predominantly in urban laboratory or hospital settings in sub-Saharan Africa, in collaboration with experts from the US and Europe, with limited data in remote rural settings in Southeast Asia. Although one evaluation study [15] was a multicenter study performed in India, China, Kenya, USA and Thailand, the validation took place in credentialed clinical laboratories with full resources and technical skills that fulfill all requirements for CD4 testing standards. Unbiased scientific evidence for any new CD4 testing technologies is needed to enable the National Health Security Office (NHSO), Thailand Ministry of Public Health, to make decisions when selecting such technologies to be used in Thailand. We, as one of the national reference laboratories, therefore conducted this study to evaluate the use of FACSPresto for determining CD4+ T-lymphocytes (both percentage and absolute count) and Hb from HIV-infected blood samples obtained from routine testing at the primary healthcare clinics where this near-patient CD4 counter would ideally be used. Comparison was with the standard three-color flow cytometric method in a well-equipped clinical laboratory at the provincial hospital. According to WHO 2013 consolidated guidelines [22], absolute CD4+ T-lymphocyte count values below or equal to 500 cells/μL are used for initiation of ART in some countries including Thailand, and because CD4 T-lymphocyte counts of less than 200 cells/µL is considered as a critical threshold for HIV patients with advanced disease, data analysis on bias and misclassification of samples with CD4 T-lymphocyte values at the thresholds of 500 and 200 cells/μL were subjected to separate statistical analysis.

## 2. Materials and Methods

### 2.1. Study Design

Evaluation of the BDB FACSPresto near-patient CD4 counter was carried out in two phases. We first carried out a feasibility study that involved evaluation of the hardware, software and other technical requirements for the system followed by comparison of the performance of the FACSPresto with the standard three-color flow cytometry-based approach using BDB Tritest™ reagent with BDB TruCOUNT™ beads (San Jose, CA, USA) and analyzed by BDB FACSCalibur™ (San Jose, CA, USA) at the Department of Immunology, Faculty of Medicine Siriraj Hospital, Mahidol University, Bangkok. This clinical laboratory has been accredited by ISO15189 since 2007 and joined the United Kingdom National External Quality Assurance Scheme (UK NEQAS) for leucocyte immunophenotyping in 2010. Phase I evaluation was part of the Thailand NHSO’s requirement for validation of the FACSPresto which has been registered for approval from the Medical Device Registration of the Thai Food and Drug Administration (FDA) before its importation into the country. This comparison was followed by Phase II which included both evaluations of the FACSPresto on HIV-infected blood samples and assessment of the performance of healthcare workers at four primary healthcare clinics affiliated with the Department of Medical Technology, Chonburi Provincial, again comparing it with standard flow cytometry at the Chonburi Hospital. The routine performance of CD4 testing at this hospital laboratory has been certified by the Thai National Program for Quality Assessment and Standardization for Flow Cytometry.

### 2.2. Patients and Blood Sample Collection

Fifty HIV-1 seropositive patients (30 males and 20 female) from the Clinic of Infectious and Immunodeficiency Diseases, Out-Patient Department, Siriraj Hospital, were recruited and evaluated in the first study phase. Two milliliters of venous blood from each patient were collected into tubes containing K3EDTA and immediately transported to the laboratory at the Department of Immunology, Faculty of Medicine Siriraj Hospital for immunophenotyping. In the second evaluation phase, venous blood samples were collected from a total of 180 HIV-infected subjects from four primary healthcare clinics. The average age was 40 years (range, 4.9–71 years) with a 1.15 male to female (107/93) ratio. Each individual blood sample was tested on the FACSPresto system by a healthcare worker at the primary healthcare clinic where it was collected. Leftover blood samples from all participating healthcare clinics were transported to Chonburi Provincial Hospital and processed for immunophenotyping and Hb determination within 6 h. Hb concentration (g/dL) in each blood sample was photocolorimetrically analyzed using SLS-HGB, a cyanide-free method in Sysmex XT-2000i automated hematology analyzer (Sysmex Corporation, Kobe, Japan).

### 2.3. Standard TriTEST/TruCOUNT Tubes Method

Staining of the peripheral blood samples was performed according to the BDB’s recommendations. The lyse-no-wash-stained blood samples were finally acquired in a FACSCalibur flow cytometer. Both percent and absolute CD4 T-lymphocytes were automatically determined by using the CD3/CD4/CD45 application of the BDB MultiSET™ software (San Jose, CA, USA).

### 2.4. BDB FACSPresto Near-Patient CD4 Counter Method

The FACSPresto is a portable CD4 counter with unit-dose disposable cartridges for directly determining percent and absolute CD4 T-lymphocytes as well as total Hb concentration in a single drop of capillary or venous blood. To obtain the results from the system, an aliquot of well mixed EDTA blood was transferred to the cartridge; the cartridge cap was then closed and placed on the workstation to allow incubation at room temperature for 18 min to 120 min. After incubation, the test-strip was removed and the cartridge was inserted into the analyzer with individual percent, absolute CD4 T-lymphocyte counts and Hb results were displayed on the screen and printed automatically within 2–3 min. 

### 2.5. Quality Control and Assay Precision

To ensure that quality control of the CD4 T-lymphocytes and Hb determination with regard to both performance of personnel and instrument at each participating laboratory was optimal and consistent, the same batch of reagents was used throughout the study at all sites. In addition, all procedures using flow cytometer or FACSPresto were performed by a single person at each site. Operators of flow cytometer at the four participating laboratories and the healthcare workers at the primary health care clinics attended a half-day training workshop on the use of FACSPresto to ensure consistency of performance. To assess the precision of CD4 testing, quality controls were performed daily for both FACSCalibur and FACSPresto according to the BDB’s recommendations. To assess the reproducibility of both the FACSCalibur and FACSPresto systems, five fresh samples of HIV-infected blood and three stabilized whole blood preparations from the CD-Check Plus CD4 Low reagent kit (Streck, Omaha, NE, USA) were used to provide data on within- and between-run variation. Calibration of Sysmex hematology analyzer was performed according to the manufacturer’s guidelines using the Sysmex recommended calibrator products.

To determine whether capillary blood samples from finger-sticks provide suitable alternatives to venipuncture samples, comparison of CD4 T-lymphocyte values tested from capillary blood samples was also conducted against the corresponding venous blood sample. Sixty-six capillary and venous blood samples of the same subjects with 15–18 samples from each of the four primary healthcare clinics were randomly collected and analyzed by FACSPresto. Venous blood samples from these 66 subjects were also transported to Department of Medical Technology, Chonburi Hospital for FACSCalibur analysis as described above. 

### 2.6. Statistical Analysis

GraphPad Prism 6 Software was used for statistical analyses and graphic. Validation of the CD4 T-lymphocytes and Hb concentration data obtained from the FACSPresto testing was performed by Passing and Bablok regression analysis [23] showing scatter diagram with regression line of measured data and obvious agreement of fitted regression line and identity line. A regression equation (y = a + bx) was also provided to reveal intercept (a) and slope (b) difference with their confidence interval of 95% (95% CI). Bland–Altman statistical bias analysis [24] was used by graphically plotting the difference between each pair of data measurements (FACSPresto—standard method) on the vertical axis against the average of the pair (FACSPresto + standard method)/2 on the horizontal axis. The average absolute difference between the two methods (the bias) and the limits of agreement (LOA; equivalent to the mean difference ± 1.96SD) were then calculated. Percent similarity of the two methods was performed by taking the average between the two methods, divided by the standard method, and multiplying by 100 [25].

## 3. Results

When operational precision for with-in run variation of the two CD4 testing systems was determined, the mean percent coefficient of variation (CV) of CD4 T-lymphocyte counts derived from running eight replicates from each of the eight samples was less than 3% (2.6 ± 1.2%) for the FACSCalibur and less than 4% (3.8 ± 2.0%) for the FACSPresto. For the between-run reproducibility test, the average CVs for eight replicates from three stabilized whole blood preparations determined during five non-consecutive days did not exceed 10% (7.8 ± 3.7% for FACSCalibur and 8.3 ± 4.6 for FACSPresto) (Table 1). 

Results from the first evaluation phase showed that the 50 HIV-infected blood samples analyzed by the two CD4 testing system gave similar percent and absolute CD4 T-lymphocyte values (Table 2). Passing and Bablok regression analyses of both percentage and absolute values from the FACSPresto showed very high correlation with the standard FACSCalibur system. The 95% CIs for intercept and slope were +0.06 to 1.82 and 0.98 to 1.06, respectively, for percentage values and were −32.17 to 36.20 and 0.97 to 1.13, respectively, for the absolute values. The mean biases from Bland–Altman bias plots of both percentage and absolute values were +1.2% and +20.5 cells/µL, respectively. 

A result comparing CD4 T-lymphocyte values from capillary blood obtained from 66 HIV-infected subjects from the four primary healthcare clinics against the corresponding venous blood analyzed either by the FACSPresto Near-Patient CD4 counter on sites or the standard TriTEST/TruCOUNT FACSCalibur system at the Chonburi Hospital showed closed agreement for both mean percentage and mean absolute CD4 T-lymphocytes (Table 3). Passing and Bablok regression analyses of both percentage and absolute values also showed good correlation results Bland–Altman biases based on mean percentage CD4 T-lymphocyte values of −1.2% and 54 cells/µL for absolute values. When capillary blood results on the FACSPresto were compared with venous blood results obtained from the FACSCalibur, the mean percentages CD4 T-lymphocytes were 27.05 ± 8.39% and 28.23 ± 8.18% with mean absolute CD4-T lymphocyte counts of 928 ± 656 and 874 ± 617 cells/µL, respectively. Passing and Bablok regression analysis for equivalency between capillary and venous blood results provided y = −0.62 + 0.97x for the percentage values and y = −34.41 + 1.09x for the absolute CD4-T-lymphocyte values. The mean percent Bland–Altman biases based on the mean percentage and the absolute values were −1.2% and 40.4 cells/µL, respectively. 

**Figure 1:**
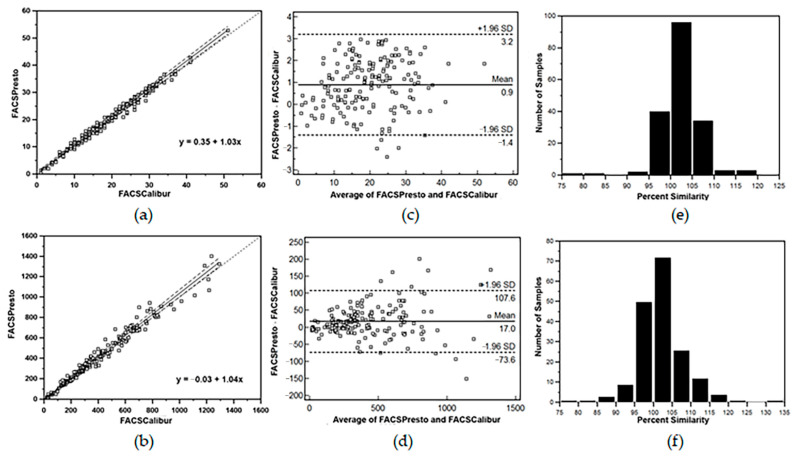
(**a**,**b**) Passing and Bablok regression analyses; (**c**,**d**) Bland–Altman bias plots; and (**e**,**f**) percent similarity plots of percent CD4 T-lymphocytes (top panel) and absolute CD4 T-lymphocyte counts (lower panel) of all 180 HIV-1 infected blood samples between the standard TriTEST/TruCount BD FACSCalibur and the BD FACSPresto near-patient CD4 counter. Passing and Bablok plots showing regression line of measured data and agreement of fitted regression line and identity line. In Bland–Altman bias plots, the horizontal lines at the center indicate the mean bias; the lower and upper lines represent LOA ± 1.96SD.

**Figure 2:**
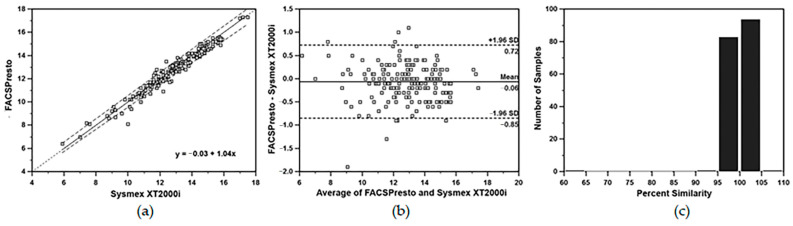
(**a**) Passing and Bablok regression analysis; (**b**) Bland–Altman bias plot; and (**c**) percent similarity plot of Hb measurements from BD FACSPresto near-patient CD4 counter and Sysmex XT2000i hematology analyzer. Passing and Bablok plot showing regression line of measured data and agreement of fitted regression line and identity line. In Bland–Altman bias plot, the horizontal line at the center indicates the mean bias; the lower and upper lines represent LOA ± 1.96SD.

When pooled data of CD4 T-lymphocyte values from 180 HIV- infected blood samples analyzed by FACSPresto near-patient CD4 Counter method from all four primary healthcare clinics were compared with the data obtained from the standard TriTEST/TruCOUNT FACSCalibur system at the Chonburi Hospital, Passing and Bablok plots from the FACSPresto system were excellent with y = 0.35 + 1.03x for all percent CD4 T-lymphocyte values (Figure 1a) and y = −0.03 + 1.04x for all absolute CD4 T- lymphocyte counts (Figure 1b). The overall mean bias, LOA and percent similarity for the CD4 T-lymphocyte data comparisons were +0.9% with LOA of −1.4% to 3.2% (Figure 1c) and +17.0 cells/µL with LOA of −73.6 to +107.6 cells/µL (Figure 1d), for percentage and absolute CD4 T-lymphocyte values, respectively. There were 7.2% (13/180) misclassified values (9 values above and 4 below) of the overall 180 CD4 T-lymphocyte results from FACSPresto compared with the FACSCalibur. The percent similarity for all percent and absolute values of CD4 T-lymphocytes were +102.5 ± 4.7 and +102.1 ± 6.4, respectively (Figure 1e,f). Passing and Bablok regression analysis of the results from Hb concentration in venous blood samples showed a good correlation with the Sysmex hematology analyzer (y = −0.03 + 1.04x). The 95% CIs for the intercept and the slope were −7.95 to 6.58 and 1.01 to +1.07, respectively. The 95% CI for intercept value indicates the systematic difference and 95% CI for slope value indicates the proportional difference between two methods. Thus, these indicated good agreement between these two methods. Bland–Altman mean % bias reported with 95% LOA showed slightly under-estimations of −0.06 g/dL (LOA = −0.85 to + 0.72 g/dL) with % similarity for all Hb concentrations of 99.6 ± 3.23 (Figure 2a–c). 

**Figure 3:**
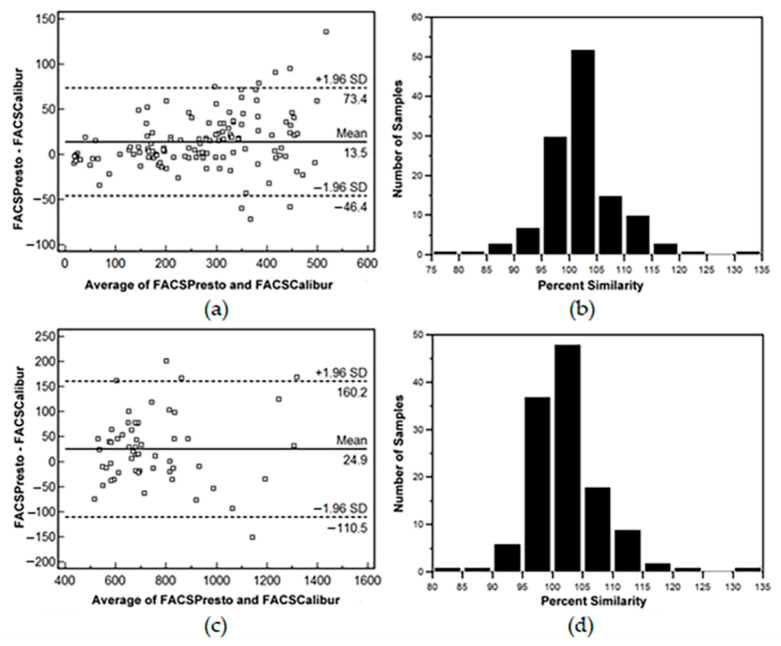
(**a**,**c**) Bland–Altman bias plots and (**b**,**d**) percent similarity plots of absolute CD4 T-lymphocyte counts of <500 cells/µL (top panel) and absolute CD4 T-lymphocyte counts > 500 cells/µL (lower panel) of HIV-1 infected blood samples between the standard TriTEST/TruCount BD FACSCalibur and the BD FACSPresto near-patient CD4 counter. In the Bland–Altman bias plots, the horizontal lines at the center indicate the mean bias; the lower and upper lines represent LOA ± 1.96SD.

**Figure 4:**
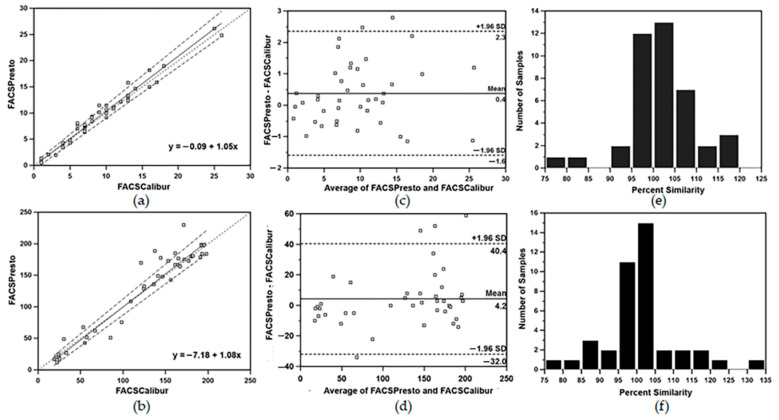
(**a**,**b**) Passing and Bablok regression analysis; (**c**,**d**) Bland–Altman bias plot and (**e**,**f**) percent similarity plot of percent CD4 T-lymphocytes (top panel) and absolute CD4 T-lymphocyte counts (lower panel) at the threshold of 200 cells/µL of HIV-1 infected blood samples between the standard TriTEST/TruCount BD FACSCalibur and the BD FACSPresto near-patient CD4 counter. Passing and Bablok plot showing regression line of measured data and agreement of fitted regression line and identity line. In Bland–Altman bias plot, the horizontal line at the center indicates the mean bias; the lower and upper lines represent LOA ± 1.96SD.

When bias analyses were separately performed on absolute CD4 T-lymphocyte counts of <500 cells/µL or >500 cells/µL, the FACSPresto gave higher values; the overall mean bias, LOA and percent similarity between the FACSPresto and the standard TriTEST/TruCOUNT FACSCalibur system were +13.5 cells/µL with LOA of −46.4 to +73.4 cells/µL and the percent similarity was +102.1 ± 71. The sensitivity and specificity of the FACSPresto was 98.4% (94.3–99.8%) and 96.4% (87.7–99.6%), respectively. A total of 6.5% (8/124) of results were misclassified when using a threshold of CD4 T-lymphocyte counts of <500 cells/µL (Figure 3a,b), and a bias value of +24.9 cells/µL with LOA of −110.5 to +160.2 cells/µL, and the percent similarity was +101.9 ± 4.7 when the values were >500 cells/µL (Figure 3c,d). Using CD4 T-lymphocyte counts of ˂200 cells/µL as a critical threshold for advanced HIV cases, FACSPresto results were comparable to those of the standard TriTEST/TruCOUNT method. Passing and Bablok regression analysis for %CD4 T-lymphocytes at this threshold showed y = −0.09 + 1.05x with 95% CI of intercept and slope of −0.77 to 0.39 and 0.98 to 1.12, respectively (Figure 4a). For absolute CD4 T-lymphocyte counts at this threshold, the Passing and Bablok regression plot showed y = −7.18 + 1.08x (Figure 4b). The mean bias comparison between the FACSPresto and the TriTEST/TruCOUNT method results was +0.4% (LOA of −1.6% to +2.3%) and + 4.2 cells/µL with LOA of −32.0 to +40.4 cells/µL for percentage and absolute CD4 T-lymphocyte values, respectively (Figure 4c,d). Four out of 41 patients, or 9.75%, were misclassified at this 200 cells/µL threshold by FACSPresto compared with the FACSCalibur. The percent similarity for percent and absolute values of CD4 T-lymphocytes were +101.8 ± 7.8 and +99.8 ± 2.5, respectively (Figure 4e,f).

## 4. Discussion

In this study, the percentage and absolute number of CD4 T-lymphocytes obtained from the FACSPresto correlated highly with those from the standard bead-based, three-color FACSCalibur system both at the ISO15189 certified university hospital’s clinical laboratory and the primary healthcare clinics. The performance outcomes for venous blood testing reported here were similar to other previous studies where venous blood sampling was used [15,16,17,19,21]. Most of these studies revealed a consistent over-estimation of percent CD4 T-lymphocyte count of +2.29% (range +0.60% to +6.90%) and +17.70 cells/µL (range −0.78 cells/µL to +44.0 cells/µL) and over-estimation for the absolute CD4 T-lymphocytes (Table 4). Like other reports, our testing with venous blood also revealed a slight over-estimation of +0.9% for percent CD4 T-lymphocyte count, with a mean absolute of +17.0 cells/ µL over-estimation for the absolute CD4 T-lymphocytes. It should be noted here that, although all studies used single-platform method for determination of CD4 T-lymphocytes, there were some different approaches in the performance evaluation of FACSPresto in these studies. While most studies [15,16,17,21] employed BD system and BD CD4 reagents, one study from South Africa used FlowCare™ PanLeuco-gated (PLG/CD4) method and analyzed on Beckman Coulter XL flow cytometer [19]. 

In our field study, the overall bias for all absolute CD4 T-lymphocyte counts was +17.0 cells/µL with 7.2% (13/180) misclassification. At thresholds of 500 and/or 200 cells/µL, the biases were +13.5 and +4.2 cells/µL with 6.5% (8/124) and 9.75% (4/41) misclassifications, respectively. These findings suggest that CD4 T-lymphocyte counts of misclassified patients at all CD4 thresholds were almost statistically similar with most of the misclassified samples had CD4 T-lymphocyte counts close to the threshold of 500 cells/µL than those at the threshold of 200 cells/µL. While these misclassifications might influence clinical decision making at the CD4 T-lymphocyte count of 500 cells/µL threshold, their impact is likely to be minimal as long as the patients are monitored by the same CD4 system and a CV is less than 10%. Despite the misclassification of some samples at the CD4 T-lymphocyte threshold of 200 cells/µL may be expected, this should pose no impact on treatment timing as patients have already received treatment if their CD4 T-lymphocyte counts are less than 500 cells/µL. 

Since the FACSPresto system is designed to be used in non-laboratory settings, such as voluntary counseling and testing (VCT) centers or primary health clinics, where infrastructure is limited and capillary blood sample collection by finger prick using lancets is therefore more realistic, our paired comparison of both percentage and absolute CD4 T-lymphocyte values in the capillary and venous blood samples showed a good correlation with an acceptable bias both with FACSPresto in the field study and the predicate FACSCalibur system at the provincial hospital level. Our results were in line with several previous studies [15,16,17] which show close agreement of the FACSPresto and the FACSCalibur in capillary and venous blood results, suggesting that the capillary blood collection can be used as an alternative way of providing capacity for rapid and cost effective CD4 T-lymphocyte testing in settings where conventional flow cytometry cannot be implemented. Although some drawbacks of using capillary from finger prick revealing lower levels of precision when analyzing by FACSPresto or other alternative POC systems, these plausibly due to varying experience of personnel in finger pricking and not the POC systems [16,19,26,27,28]. 

Many evaluation studies, including those performed by our laboratory, have shown that a number of POC systems other than the FACSPresto CD4 testing system performed well in comparison with the standard bead-based, flow cytometric methods [29,30,31,32]. However, the operation of some of these POC systems is in fact quite complicated and still requires highly trained personnel. FACSPresto, on the other hand, is simple, easy to use and requires minimal training and can be operated by para-medical staff such as nurses and health volunteers at the primary healthcare clinics where trained laboratory personnel are not available. Apart from CD4 testing, FACSPresto also provides Hb determination which would be useful for monitoring anemic condition in HIV-infected patients during pregnancy. In addition, such a system utilizes an off-board reaction which allows more samples to be tested per day, which is particularly important in a busy rural clinic. Throughput is conservatively estimated to be 10 cartridges per hour, with around 23 min from blood application to reporting of results. In addition, the cartridges use dried-down fluorescently labeled MoAbs that do not need cold chain storage, a very useful and practical feature in tropical countries. It should also be noted that there were approximately 2.6% of the total tested blood samples (six of 230 samples from the two evaluation phases) that had to be repeated, either by a second acquisition of the same cartridge or by a new preparation of blood sample using a new cartridge. This repeated analysis of samples is not only inconvenient to the operator, but will likely impact on the hospital’s healthcare budget which is under pressure to operate in a cost-efficient manner.

## 5. Conclusions

The BD FACSPresto near-patient CD4 counter provides both percentage and absolute CD4 T-lymphocyte values which are in good agreement with results from the standard bead-based, flow cytometric system. Compared to other POC systems, FACSPresto has the advantage of having the complete incubation process outside the system and thus making a relatively high sample throughput. Such system can be operated by the para-medical staff after minimum training. The operators at the four primary healthcare clinics found the system to be easy to use after a half day of training. This near-patient CD4 counter should therefore not only provide research-based evidence on the system’s performance to enable the Ministry of Public Health to make decision when selecting such technology to be implemented in Thailand, but also improve HIV management by facilitating wider access to CD4 testing, increasing timely ART services, and reducing patient loss to follow-up at the primary healthcare settings in Thailand and other resource-limited countries.

## Figures and Tables

**Table 1:** Reproducibility of the BD FACSCalibur and the BD FACSPresto system determined by analysis of 8 replicates from one fresh HIV-1 infected blood sample and 8 replicates from a stabilized CD-Check Plus Low blood.

Variation	Mean % CV ± SD	
	FACSCalibur/TriTest/TruCount	FACSPresto
Within-run	2.6 ± 1.2	3.8 ± 2.0
Between-run	7.8 ± 3.7	8.3 ± 4.6

**Table 2:** Comparison analysis of percent and absolute CD4 T-lymphocyte values between the standard TriTEST/TruCount BD FACSCalibur and the BD FACSPresto near-patient CD4 counter at the ISO15189-accredited clinical laboratory by using Passing and Bablok regression and Bland–Altman mean bias analysis.

Parameter	CD4 (%)	Absolute CD4 (cells/μL)
Regression	y = 0.75 + 1.02x	y = 0.92 + 1.07x
95% * CI for intercept	+0.06 to 1.82	−32.17 to 36.20
95% CI for slope	0.98 to 1.06	0.97 to 1.13
Bland–Altman mean bias	+1.2	+20.5
** LOA (mean difference ± 1.96SD)	1.0 to 3.3%	−136.1 to +177.2

* CI; Confidence Interval; ** LOA; Limits of Agreement.

**Table 3:** Comparison analysis of percent and absolute CD4 T-lymphocyte values between capillary and venous blood of the same 66 HIV-infected blood samples on BD FACSPresto near-patient CD4 counter at the primary healthcare clinics, and on BD FACSCalibur at the ISO15189-accredited clinical laboratory by using Passing and Bablok regression and Bland–Altman mean bias analysis.

	**FACSPresto Venous vs. Capillary**	
**Parameter**	**CD4 (%)**	**Absolute CD4 (cells/μL)**
Mean		
Venous blood	28.23 ± 8.18%	874 ± 617
Capillary blood	27.05 ± 8.39%	928 ± 656
Regression	y = −1.34 + 1.01x	y = −15.84 + 1.91x
95% * CI for intercept	−2.67 to 0.03	−48.77 to 9.46
95% CI for slope	0.97 to 1.06	1.05 to 1.14
Bland–Altman mean bias	−1.2%	+54
** LOA (mean difference ± 1.96SD)	−4.6 to 2.2%	−148.3 to +256.2
	**FACSPresto Capillary vs. FACSCalibur**	
**Parameter**	**CD4 (%)**	**Absolute CD4 (cells/μL)**
Regression	y = −0.62 + 0.97x	y = −34.41 + 1.09x
95% * CI for intercept	−2.11 to 0.57	−73.26 to 4.82
95% CI for slope	0.93 to 1.04	1.03 to 1.15
Bland–Altman mean bias	−1.2%	+40.4
** LOA (mean difference ± 1.96SD)	−5.6 to 3.2%	−273.6 to +354.4

* CI; Confidence Interval; ** LOA; Limits of Agreement.

**Table 4:** Comparison analysis of values from the present study with previously published data for the performance evaluation of BD FACSPresto near-patient CD4 counter with the standard flow cytometer using venous blood samples.

Parameter	Sample Size	Flow Cytometer	Method	Bland–Altman Mean Bias Analysis (95% CI)	
				% CD4	Absolute CD4 (cells/µL)
Present study	180	BD FACSCalibur	TriTEST/ TruCOUNT	+0.9 (−1.4 to 3.2)	+17.0 (−73.6 to 107.6)
South Africa [19]	214	BC Coulter XL	FlowCare PanLeuco-gate	+1.41 (−1.7 to 4.5)	+40.39 (−49.4 to 130.2)
Kenya/USA [17]	189	BD FACSCalibur	TriTEST/ TruCOUNT	+3.19 (−14.01 to 20.40)	−0.78 (−21.26 to 19.69)
Tanzania/Belgium [16]	200	BD FACSCalibur	TriTEST/ TruCOUNT	+0.6 (−11 to 13)	+2.5 (−15 to 20)
India/China/Kenya/USA/Thailand [15]	720	BD FACSCalibur	TriTEST/ TruCOUNT	+0.75 (−2.12 to 3.61)	+3.10 (−22.89 to 16.80)
Ghana [21]	53	BD FACSCount	new CD4 reagent	+6.9 (−11.9 to 25.7)	+44 (−72 to 160)

## Data Availability

The data presented in this study are available on request from the corresponding author.

## References

[1] Belaunzaran-Zamudio P.F., Caro-Vega Y.N., Shepherd B.E., Rebeiro P.F., Crabtree-Ramirez B.E., Cortes C.P., Grinsztejn B., Gotuzzo E., Mejia F., Padgett D. (2020). The population impact of late presentation with advanced HIV disease and delayed antiretroviral therapy in adults receiving HIV care in Latin America. Am. J. Epidemiol..

[2] Brennan A.T., Maskew M., Larson B.A., Tsikhutsu I., Bii M., Vezi L., Fox M.P., Venter W.D., Ehrenkranz P., Rosen S. (2019). Who is seeking antiretroviral treatment for HIV now? Characteristics of patients presenting in Kenya and South Africa in 2017–2018. J. Int. AIDS Soc..

[3] Auld A.F., Shiraishi R.W., Oboho I., Ross C., Bateganya M., Pelletier V., Dee J., Francois K., Duval N., Antoine M. (2017). Trends in prevalence of advanced HIV disease at antiretroviral therapy enrollment–10 countries, 2004–-2015. MMWR Morb. Mortal. Wkly. Rep..

[4] UNAIDS Understanding Fast-Track: Accelerating Action to End the Aids Epidemic by 2030. Unaids.org/sites/default/files/media_asset/201506_JC2743_Understanding_FastTrack_en.pdf.

[5] UNAIDS The Joint United on Nations Programme HIV/AIDS. https://www.unaids.org/en/regionscountries/countries/thailand.

[6] World Health Organization Antiretroviral Therapy for HIV Infection in Adults and Adolescents: Recommendations for a Public Health Approach—2010 Revision, 2010 Rev. https://apps.who.int/iris/handle/10665/43554.

[7] Fauci A.S., Macher A.M., Longo D.L., Lane H.C., Rook A.H., Masur H., Gelmann E.P. (1984). NIH conference. Acquired immunodeficiency syndrome: Epidemiologic, clinical, immunologic, and therapeutic considerations. Ann. Intern. Med..

[8] HIV Surrogate Marker Collaborative Group (2000). Human immunodeficiency virus type 1 RNA level and CD4 count as prognostic markers and surrogate end points: A meta-analysis. HIV Surrogate Marker Collaborative Group. AIDS Res. Hum. Retrovir..

[9] World Health Organization (2021). Consolidated Guidelines on HIV Prevention, Testing, Treatment, Service Delivery and Monitoring: Recommendations for a Public Health Approach.

[10] Ford N., Meintjes G., Vitoria M., Greene G., Chiller T. (2017). The evolving role of CD4 cell counts in HIV care. Curr. Opin. HIV AIDS.

[11] (1997). 1997 revised guidelines for performing CD4+ T-cell determinations in persons infected with human immunodeficiency virus (HIV). Centers for Disease Control and Prevention. MMWR Recomm. Rep..

[12] (1998). National Committee for Clinical Laboratory Standards, Clinical Application of Flow Cytometry: Immunophenotyping of Lymphocytes. Approved Guideline.

[13] Mandy F.F., Nicholson J.K., McDougal J.S., CDC (2003). Guidelines for performing single-platform absolute CD4+ T-cell determinations with CD45 gating for persons infected with human immunodeficiency virus. Centers for Disease Control and Prevention. MMWR Recomm. Rep..

[14] BD Biosciences, Becton Dickinson FACSPresto^TM^ System: A Near-Patient Complete CD4 Testing Solution. https://www.bd.com/documents/bd-legacy/brochures/biosciences/BDB_BD-FACSPresto-TS_TR.pdf.

[15] Thakar M., Angira F., Pattanapanyasat K., Wu A.H.B., O’Gorman M., Zeng H., Qu C., Mahajan B., Sukapirom K., Chen D. (2017). CD4 Lymphocyte Enumeration and Hemoglobin Assessment Aid for Priority Decisions: A Multisite Evaluation of the BD FACSPresto™ System. Open AIDS J..

[16] Daneau G., Aboud S., Prat I., Urassa W., Kestens L. (2017). Performance of FACSPresto Point-of-Care Instrument for CD4-T Cell Enumeration in Human Immunodeficiency Virus (HIV)-Infected Patients Attending Care and Treatment Clinics in Belgium and Tanzania. PLoS ONE.

[17] Angira F., Akoth B., Omolo P., Opollo V., Bornheimer S., Judge K., Tilahun H., Lu B., Omana-Zapata I., Zeh C. (2016). Clinical Evaluation of the BD FACSPresto Near-Patient CD4 Counter in Kenya. PLoS ONE.

[18] Makadzange A.T., Bogezi C., Boyd K., Gumbo A., Mukura D., Matubu A., Ndhlovu C.E. (2016). Evaluation of the FACSPresto, a New Point of Care Device for the Enumeration of CD4% and Absolute CD4+ T Cell Counts in HIV Infection. PLoS ONE.

[19] Coetzee L.M., Moodley K., Glencross D.K. (2016). Performance Evaluation of the Becton Dickinson FACSPresto Near-Patient CD4 Instrument in a Laboratory and Typical Field Clinic Setting in South Africa. PLoS ONE.

[20] Negedu-Momoh O.R., Jegede F.E., Yakubu A., Balogun O., Abdullahi M., Badru T., Oladele E.A., Agbakwuru C., Khamofu H., Torpey K. (2017). Performance evaluation of BD FACSPresto point of care CD4 analyzer to enumerate CD4 counts for monitoring HIV infected individuals in Nigeria. PLoS ONE.

[21] Moran Z., Sacks J.A., Frimpong F.K., Frimpong A.B., Ben Amor Y. (2019). Performance of the BD-FACS Presto for CD4 count and hemoglobin measurement in a district hospital and rural laboratory in Ghana. PLoS ONE.

[22] World Health Organization March 2014 Supplement to the 2013 Consolidated Guidelines on the Use of Antiretroviral Drugs for Treating and Preventing HIV Infection: Recommendations for a Public Health Approach. https://apps.who.int/iris/handle/10665/104264..

[23] Passing H., Bablok (1983). A new biometrical procedure for testing the equality of measurements from two different analytical methods. Application of linear regression procedures for method comparison studies in clinical chemistry, Part I. J Clin. Chem. Clin. Biochem..

[24] Bland J.M., Altman D.G. (1986). Statistical methods for assessing agreement between two methods of clinical measurement. Lancet.

[25] Scott L.E., Galpin J.S., Glencross D.K. (2003). Multiple method comparison: Statistical model using percentage similarity. Cytom. B Clin. Cytom..

[26] Mtapuri-Zinyowera S., Chideme M., Mangwanya D., Mugurungi O., Gudukeya S., Hatzold K., Mangwiro A., Bhattacharya G., Lehe J., Peter T. (2010). Evaluation of the PIMA point-of-care CD4 analyzer in VCT clinics in Zimbabwe. J. Acquir. Immune Defic. Syndr..

[27] Scott L.E., Campbell J., Westerman L., Kestens L., Vojnov L., Kohastsu L., Nkengasong J., Peter T., Stevens W. (2015). A meta-analysis of the performance of the Pima CD4 for point of care testing. BMC Med..

[28] Glencross D.K., Coetzee L.M., Faal M., Masango M., Stevens W.S., Venter W.F., Osih R. (2012). Performance evaluation of the Pima point-of-care CD4 analyser using capillary blood sampling in field tests in South Africa. J. Int. AIDS Soc..

[29] Dieye T.N., Vereecken C., Diallo A.A., Ondoa P., Diaw P.A., Camara M., Karam F., Mboup S., Kestens L. (2005). Absolute CD4 T-cell counting in resource-poor settings: Direct volumetric measurements versus bead-based clinical flow cytometry instruments. J. Acquir. Immune Defic. Syndr..

[30] Pattanapanyasat K., Lerdwana S., Noulsri E., Chaowanachan T., Wasinrapee P., Sakulploy N., Pobkeeree V., Suksripanich O., Thanprasertsuk S., Spira T.J. (2005). Evaluation of a new single-parameter volumetric flow cytometer (CyFlow(green)) for enumeration of absolute CD4+ T lymphocytes in human immunodeficiency virus type 1-infected Thai patients. Clin. Diagn. Lab. Immunol..

[31] Pattanapanyasat K., Phuang-Ngern Y., Lerdwana S., Wasinrapee P., Sakulploy N., Noulsri E., Thepthai C., McNicholl J.M. (2007). Evaluation of a single-platform microcapillary flow cytometer for enumeration of absolute CD4+ T-lymphocyte counts in HIV-1 infected Thai patients. Cytom. B Clin. Cytom..

[32] Sukapirom K., Onlamoon N., Thepthai C., Polsrila K., Tassaneetrithep B., Pattanapanyasat K. (2011). Performance evaluation of the Alere PIMA CD4 test for monitoring HIV-infected individuals in resource-constrained settings. J. Acquir. Immune Defic. Syndr..

